# A Dead Reckoning Calibration Scheme Based on Optimization with an Adaptive Quantum-Inspired Evolutionary Algorithm for Vehicle Self-Localization

**DOI:** 10.3390/e24081128

**Published:** 2022-08-15

**Authors:** Biao Yu, Hui Zhu, Deyi Xue, Liwei Xu, Shijin Zhang, Bichun Li

**Affiliations:** 1 Hefei Institutes of Physical Science, Chinese Academy of Sciences, Hefei 230031, China; 2 Department of Mechanical and Manufacturing Engineering, University of Calgary, Calgary, AB T2N 1N4, Canada

**Keywords:** adaptive quantum-inspired evolutionary algorithm, dead reckoning, intelligent vehicle, optimization, parameter calibration

## Abstract

Parameter calibration is critical for self-localization based on dead reckoning in the control of intelligent vehicles such as autonomous driving. Most traditional calibration methods for robotics control based on dead reckoning rely on data collection with specially designed paths. For the calibration of parameters in the control of intelligent vehicles, the design of such paths is considered impossible due to the complexity of road conditions. To solve this problem, an optimization-based dead reckoning calibration scheme is introduced in this research using the differential global positioning system to obtain the actual positions of the intelligent vehicle. In this scheme, the difference between the positions obtained through dead reckoning and the positions obtained through the differential global positioning system is selected as the optimization objective function to be minimized. An adaptive quantum-inspired evolutionary algorithm is developed to improve the quality and efficiency of optimization. Experiments with an intelligent vehicle were also conducted to demonstrate the effectiveness of the developed calibration scheme. In addition, the newly introduced adaptive quantum-inspired evolutionary algorithm is compared with the classic genetic algorithm and the classic quantum-inspired evolutionary algorithm using eight benchmark test functions considering computation quality and efficiency.

## 1. Introduction

The development of intelligent vehicles, particularly autonomous cars, has attracted considerable attention from both academics and industries. Without human operations, an intelligent vehicle can be driven autonomously in various complex environments by perceiving the surrounding world with its onboard sensors such as laser radars, cameras, and navigation and localization devices. A highly accurate and reliable localization service plays an important role for an autonomous car. Unfortunately, the existing vehicle localization techniques still cannot fully satisfy these requirements.

Presently, localization using the Global Positioning System (GPS) is the most widely used method. The positioning accuracy of civilian GPS can reach 15–20 m (95%) after the USA government removed the selective availability constraints in 2000 [1]. In this method, however, signals of at least four satellites need to be received synchronously and the GPS receiver requires an unobstructed view of the sky. Therefore, the accuracy of GPS can be degraded severely when the receiver works in poor signal areas such as underpasses, high buildings, tunnels, and city canyons [2]. A real-world field test conducted in London showed that GPS positioning errors in some cases could be offset from the true position by more than 50 m [3]. In order to overcome the limitations of GPS technology, the dead reckoning (DR) method is often used to provide localization services for vehicles without using external observations. Dead reckoning can be used to obtain the positions of a vehicle by the information obtained from wheel encoders (odometer), inertial navigation systems [4,5], LIDAR [6], or cameras [7]. However, due to the quality in the measurements with vehicle sensors, both systematic errors and random errors are brought into DR. The accuracy of DR localization can be degraded significantly after a period of time. In general, the random errors, which are mainly caused by rough ground and wheel slippages, can be reduced by combining DR and other sensors such as GPS and gyroscopes [8,9,10,11]. Systematic errors are usually caused by manufacturing and installation errors of the odometry sensors. For intelligent vehicles, the systematic errors are the major error sources when it runs on smooth road surfaces [6]. In this research, only systematic errors are considered in the calibration scheme for DR localization.

Considerable methods have been developed in the past to reduce DR systematic errors. In this research area, Borenstein and Feng [4] introduced an effective calibration scheme called UMBmark for measuring and compensating systematic odometry errors in two-wheeled differential drive mobile robots. In this scheme, the robot was programmed to travel along a path in a square of 4 by 4 m^2^, and the errors between initial and final positions were used to calibrate the robot kinematic parameters. Antonelli et al. [12] developed a method based on the least-squares technique to calibrate the odometer in a differential drive robot. In this method, the kinematic equations were defined as linear relations between the unknown parameters and the motion inputs to the odometer’s measurements, and then the least-squares method was applied to find the optimal calibration parameters. Lee et al. [13] extended the UMBmark to two-wheel differential mobile robots by considering the coupled effect of diameter and wheelbase errors, and developed a new guideline for the calibration experiments. For a car-like mobile robot, Lee et al. [14] developed a method for the calibration of tread and wheel diameters using erroneous ending positions obtained from odometry. Dogruer [15] developed a multiple model adaptive estimation (MMAE) algorithm and a least squares estimation (LSE) method to estimate the unknown parameters of an odometer. McKerrow and Ratner [16] introduced a scheme to calibrate the kinematic parameters of a four-wheel robot by using an odometer and ultrasonic range sensors. Paulo and Bijan [17] developed a method for odometer calibration and error propagation identification in the control of mobile robots using a laser interferometer. Martinelli [18] introduced a calibration approach for identifying two parameters that characterize the translational and rotational systematic components for a mobile robot with a synchronous-drive system. Lundquist et al. [19] developed a particle filter-based method to estimate the tire radii which were considered crucial for dead reckoning-based positioning. Li et al. [20] proposed a navigation map-aided dead reckoning correction method to obtain precise positioning information for advanced driving assistance systems. Takeyama et al. [7] proposed the method of using the time-series tightly coupled integration of satellite Doppler shift and IMU to estimate the heading error for visual odometry-based dead reckoning. Belhajem et al. [21] presented a method which combined the extended Kalman filter (EKF) with machine learning techniques, neural networks, or support vector machines to improve the accuracy of vehicle position estimation. Our previous research also presented a dead reckoning error correction scheme based on the extended Kalman filter and map matching [22].

Although considerable results have been achieved, all these developed methods introduced above are effective for small mobile robots with special kinematic models. In these methods, the robots were programmed to move along predefined test tracks in calibration experiments. For a real-size intelligent vehicle, however, it is impossible to drive it along a predefined road path due to the large errors in the vehicle’s mechanical systems. In this research, the differential global positioning system (DGPS) is used to get the actual positions of the driving path, and an optimization-based DR calibration scheme is introduced to reduce the systematic errors associated with the DR localization. In this optimization model, the diameter of the left rear wheel, diameter of the right rear wheel, and heading of the gyroscope are considered as the calibration parameters to reduce the localization errors in the DR. An adaptive quantum-inspired evolutionary algorithm (AQIEA) is developed to improve the optimization quality and efficiency. Two road tests with different scales were conducted to demonstrate the effectiveness of the developed method. In addition, the newly developed adaptive quantum-inspired evolutionary algorithm (AQIEA) is compared with the classic genetic algorithm (CGA) and the classic quantum-inspired evolutionary algorithm (CQIEA) using eight benchmark test functions. The results show that the new AQIEA can provide the best computation quality and efficiency among the three algorithms.

The rest of this paper is organized as follows. The optimization-based dead reckoning calibration scheme is introduced in Section 2. The adaptive quantum-inspired evolutionary algorithm is presented in Section 3. Parameter calibration for an intelligent vehicle through experiments based on the developed AQIEA for DR is explained in Section 4. A comparison among the three optimization algorithms is presented in Section 5. Conclusions are provided in Section 6.

## 2. An Optimization-Based Dead Reckoning Calibration Scheme

### 2.1. Dead Reckoning

**Figure 1:**
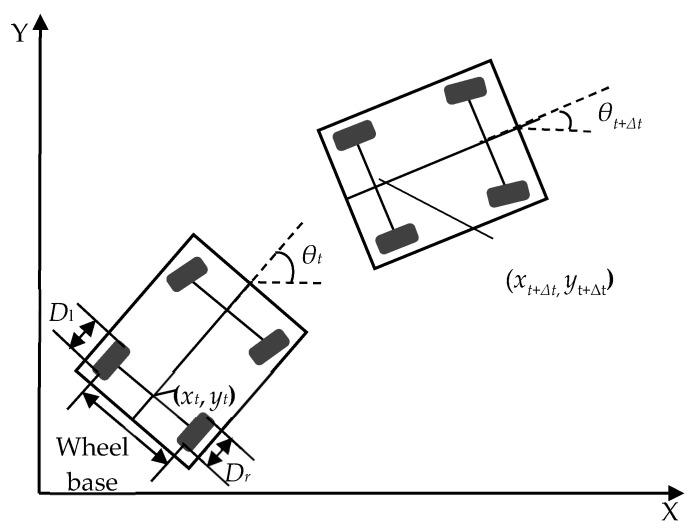
The dead reckoning localization process.

Figure 1 shows the kinematic model of a real-size IV on a 2D plane. With a given initial start position (usually obtained through a GPS), DR can be used to estimate the vehicle’s momentary positions based on the heading and displacement information obtained from on-board sensors. In this research, the DR sensors include a gyroscope and two wheel encoders for obtaining heading information and displacement information, respectively.

For the sampling period of *t*, the DR position of the vehicle can be calculated by

(1) $$\left\{\begin{array}{c} x_{t+\Delta t}=x_{t}+\Delta d\cos(\theta_{t}+\Delta \theta /2)\\ y_{t+\Delta t}=y_{t}+\Delta d\sin(\theta_{t}+\Delta \theta /2)\\ \theta_{t+\Delta t}=\theta_{t}+\Delta \theta \end{array}\right.$$

where *x_t_*, *y_t_*, and *θ_t_* are the coordinates of the vehicle along the *x*-direction and *y*-direction in the Cartesian coordinate system and the heading of the vehicle at time *t*, respectively. ∆*d* is the displacement during the sampling period between *t* and *t* + ∆*t*, calculated by

(2) $$\Delta d=\frac{\Delta d_{r}+\Delta d_{l}}{2}$$

where ∆*d_r_*_(*l*)_ is the incremental travel displacement for the right (left) rear wheel during the sampling period. ∆*d_r_*_(*l*)_ is calculated by

(3) $$\Delta d_{r(l)}=\frac{\Delta N_{r(l)}}{R_{r(l)}}\pi D_{r(l)}$$

where ∆*N_r_*_(*l*)_ is the number of increment encoder pulses of the right (left) rear wheel during the sampling period, *R_r_*_(*l*)_ is the encoder resolution of the right (left) rear wheel in the unit of pulses per revolution, and *D_r_*_(*l*)_ is the nominal diameter of right (left) rear wheel.

∆*θ* is the heading change during the sampling period calculated by

(4) $$\Delta \theta =w_{t}\Delta t$$

where *w_t_* is the angle change rate of the vehicle at time *t* measured by an onboard gyroscope.

### 2.2. Modeling of Systematic Errors for Dead Reckoning

As mentioned before, two kinds of errors, systematic errors and random errors, influence the accuracy of dead reckoning. The sources leading to systematic errors include differences between wheel diameters, deviations of wheel diameters from nominal diameters, the misalignment of wheels, and so on. The random errors usually come from the uncertainties about the effective wheelbase, the limited resolution and sampling rate of encoders, and influences of the driving environment, such as a rough road surface, wheel slippage, and so on.

When the vehicle is driven on the road, the various dead reckoning errors can be described as follows:

(5) $$\left\{\begin{array}{l} \Delta {\tilde{d}}_{r}=\Delta d_{r}+\varepsilon_{1}\\ \Delta {\tilde{d}}_{l}=\Delta d_{l}+\varepsilon_{2}\\ {\tilde{\theta }}_{t}=\theta_{t}+\varepsilon_{3} \end{array}\right.$$

where *ε*_1_ and *ε*_2_ are the errors that, respectively, need to be compensated for the incremental displacements of the right rear wheel and left rear wheel, and *ε*_3_ is the error that needs to be compensated for the heading of the vehicle. Unlike the stochastic nature of random errors, the systematic errors are determined from the geometry of the specific vehicle and they usually do not change during the vehicle’s motion. Therefore, the systematic errors can be compensated through the parameter calibration process. According to the kinematic model of the intelligent vehicle, three dominant systematic error sources are identified, i.e., the differences between the actual wheel diameters and the nominal diameters, and the gyroscope’s initial installation error due to misalignment of the gyroscope with the longitudinal axis of the vehicle. Thus, the DR systematic errors of the vehicle can be described as follows:

(6) $$\left\{\begin{array}{l} \Delta {\tilde{d}}_{r}=\Delta d_{r}+c_{1}\\ \Delta {\tilde{d}}_{l}=\Delta d_{l}+c_{2}\\ {\tilde{\theta }}_{t}=\theta_{t}+c_{3} \end{array}\right.$$

where *c*_1_ and *c*_2_ are the parameters to compensate the diameters of the right and left rear wheels, respectively, and *c*_3_ is the parameter to compensate the misalignment of the gyroscope with the longitudinal axis of the vehicle. It should be noted that when the vehicle wheels are replaced or the gyroscope is reinstalled, the calibration process should be conducted again.

After calibration, Equation (1) can be rewritten as follows:

(7) $$\left\{\begin{array}{l} {\tilde{x}}_{t+\Delta t}={\tilde{x}}_{t}+\frac{\Delta {\tilde{d}}_{r}+\Delta {\tilde{d}}_{l}}{2}\cdot \cos({\tilde{\theta }}_{t}+\Delta \tilde{\theta }/2)\\ {\tilde{y}}_{t+\Delta t}={\tilde{y}}_{t}+\frac{\Delta {\tilde{d}}_{r}+\Delta {\tilde{d}}_{l}}{2}\cdot \sin({\tilde{\theta }}_{t}+\Delta \tilde{\theta }/2)\\ {\tilde{\theta }}_{t+\Delta t}={\tilde{\theta }}_{t}+\Delta \theta \end{array}\right.$$

### 2.3. Optimization Model for Parameter Calibration in Dead Reckoning

**Figure 2:**
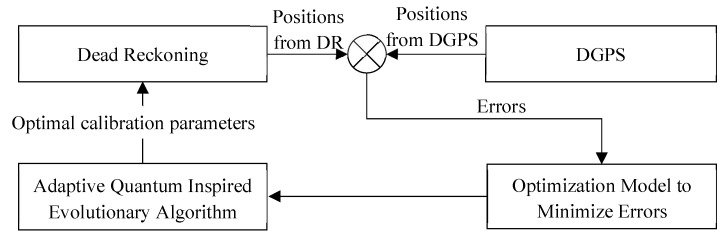
Optimization-based dead reckoning calibration scheme.

Figure 2 gives the process of parameter calibration based on the proposed dead reckoning calibration method. It should be noted that the differential GPS (DGPS) is only used for the calibration process and the initialization of DR localization.

To calibrate DR, the positioning data obtained from a high accuracy DGPS are used as the correct positions. The difference between the data from the DGPS and DR is considered as the positioning error, and it is calculated by

(8) $$\begin{array}{l} E_{i}(c_{1},c_{2},c_{3},p_{i}^{GPS},p_{i}^{DR})\\ =\sqrt{{\left(x_{i}^{GPS}-{\tilde{x}}_{i}\right)}^{2}+{\left(y_{i}^{GPS}-{\tilde{y}}_{i}\right)}^{2}}\;\;i=1,2,\cdots ,N \end{array}$$

where *E_i_* is the difference between the DR’s estimated position $p_{i}^{DR}$ and the DGPS’s position $p_{i}^{GPS}$ at the *i*-th time point, and *N* is the total number of sample points.

To identify the optimal calibration parameters of DR, an optimization model is formulated as:

(9) $$\begin{array}{l} \underset{w.r.t.c_{1},c_{2},c_{3}}{\min}M\\ s.t.\\ \left\{\begin{array}{l} c_{1}^{L}\leq c_{1}\leq c_{1}^{U}\\ c_{2}^{L}\leq c_{2}\leq c_{2}^{U}\\ c_{3}^{L}\leq c_{3}\leq c_{3}^{U} \end{array}\right. \end{array}$$

where $c_{1}^{L}$, $c_{1}^{U}$, $c_{2}^{L}$, $c_{2}^{U}$, $c_{3}^{L}$, and $c_{3}^{U}$ are the lower and upper limits of *c*_1_, *c*_2_, and *c*_3_, respectively, and *M* is the optimization objective function related to positioning errors to be minimized. The ranges of *c*_1_, *c*_2_, and *c*_3_ are usually determined by real measurements; in this study, their ranges were set as [−1, 1], [−1, 1], and [−2, 2], respectively. To identify the optimal calibration parameters, two different optimization objective functions are tested. They are the maximum positioning error and the mean positioning error considering all the positioning data:

(10) $$M=\underset{w.r.t.i}{\max}E_{i}\;\mathrm{or}\;M=\frac{1}{N}\sum_{i=1}^{N}E_{i}$$

## 3. Optimization Based on an Adaptive Quantum-Inspired Evolutionary Algorithm

### 3.1. Process of the Adaptive Quantum-Inspired Evolutionary Algorithm

**Figure 3:**
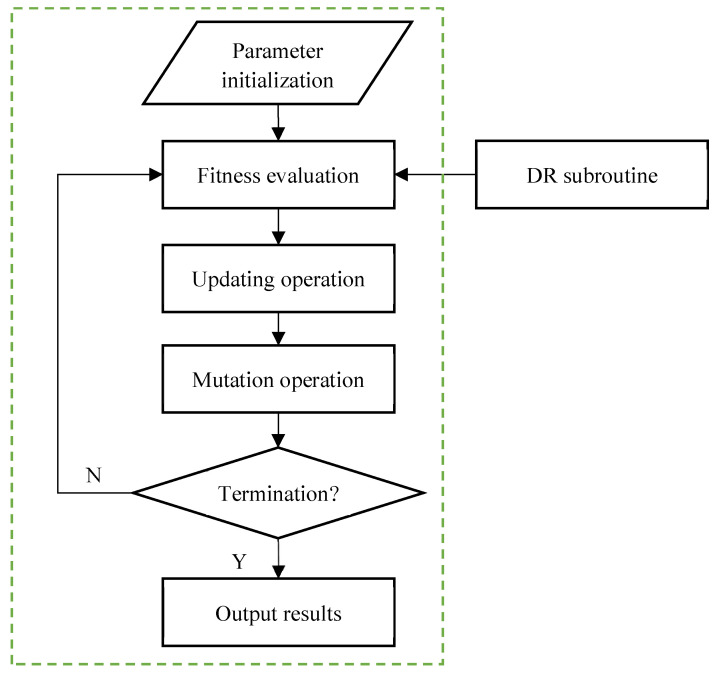
Optimization process based on AQIEA.

In this work, an adaptive quantum-inspired evolutionary algorithm (AQIEA) is introduced to solve the optimization problem defined in Equation (9). Figure 3 gives the flowchart of the AQIEA. In this algorithm, first a population of solutions is initialized. Each individual in this population is evaluated based on the fitness measure which is defined by the optimization objective function. In this work, a DR subroutine is used to calculate the optimization objective function evaluation measure. The individuals in the population are subsequently evolved using updating operations and mutation operations. When the optimization criteria are satisfied, the best individual in the population is selected as the optimal solution. Otherwise, the individuals need to be evolved again to generate better solutions.

### 3.2. Classic Quantum-Inspired Evolutionary Algorithm

The quantum-inspired evolutionary algorithm (QIEA) was introduced to improve optimization capabilities and has been employed to solve many different types of problems such as the 0–1 knapsack problem [23], stochastic job shop scheduling problem [24], neuro-fuzzy controller design problem [25], and function optimization problem [26].

QIEA was developed based on the principles and concepts in quantum computing, such as qubits and the superposition of states. In QIEA, qubit chromosomes, instead of binary, numeric, or symbolic chromosomes, are used to model optimization solutions. A qubit is the basic information unit in a two-state quantum computer. A qubit might be in the state $|0\rangle$ or state $|1\rangle$, or in any linear superposition of the two states because of the advantage that a qubit can represent a linear superposition of state $|0\rangle$ and state $|1\rangle$. Here, $|•\rangle$ is the Dirac bracket notation.

A qubit is defined as:

(11) |φ〉=α|0〉+β|1〉

where |φ〉 is a qubit state, and *α* and *β* are complex numbers to specify the probability amplitudes of the states $|0\rangle$ and $|1\rangle$, respectively. |*α*|^2^ is the probability of outcome $|0\rangle$, and |*β*|^2^ is the probability of outcome $|1\rangle$. The *α* and *β* satisfy the following condition:

(12) $$|\alpha {|}^{2}+|\beta {|}^{2}=1$$

If a system has *m* qubits, the system can present 2*^m^* states at the same time. However, in the act of observing the *m* qubits, the outcome is a single state. In the QIEA, an individual is composed of *m* qubits, and one qubit is defined by a pair of probability amplitudes $\left[\begin{array}{c} \alpha \\ \beta \end{array}\right]$. Thus, an individual with *m* qubits is defined by

(13) $$q=\left[\left.\begin{array}{c} \alpha_{1}\\ \beta_{1} \end{array}\right|\left.\begin{array}{c} \alpha_{2}\\ \beta_{2} \end{array}\right|\left.\begin{array}{c} \cdots \\ \cdots \end{array}\right|\left.\begin{array}{c} \alpha_{i}\\ \beta_{i} \end{array}\right|\left.\begin{array}{c} \cdots \\ \cdots \end{array}\right|\begin{array}{c} \alpha_{m}\\ \beta_{m} \end{array}\right]$$

where *q* is an individual in the population, and ${\left|\alpha_{i}\right|}^{2}+{\left|\beta_{i}\right|}^{2}=1$, *i* = 1, 2, …, *m*. The advantage of this representation is that an individual can represent any superposition of states. For example, an individual with three qubits can be represented as follows:

(14) $$\left[\left.\begin{array}{c} \frac{1}{\sqrt{2}}\\ \frac{1}{\sqrt{2}} \end{array}\right|\left.\begin{array}{c} \frac{1}{2}\\ \frac{\sqrt{3}}{2} \end{array}\right|\begin{array}{c} \frac{\sqrt{3}}{2}\\ \frac{1}{2} \end{array}\right]$$

It can be used to represent eight different states in this system: $|000\rangle$, $|001\rangle$, $|010\rangle$, $|011\rangle$, $|100\rangle$, $|101\rangle$, $|110\rangle$, and $|111\rangle$, and probabilities of these states are calculated as: $\frac{3}{32}$, $\frac{1}{32}$, $\frac{9}{32}$, $\frac{3}{32}$, $\frac{3}{32}$, $\frac{1}{32}$, $\frac{9}{32}$, and $\frac{3}{32}$, respectively. After decoding the qubits to classic bits, the real values can be obtained by

(15) $$x=x^{L}+\frac{{\left(b_{1}b_{2}b_{3}\ldots b_{n}\right)}_{decimal}}{2^{n}-1}(x^{U}-x^{L})$$

where *x* is the decoded real value, *x^U^* and *x^L^* are its lower and upper bounds, *n* is the length of the qubits for *x*, and (*b*_1_*b*_2_*b*_3_…*b_n_*)*_decimal_* is decimal value of the classic bits (*b*_1_*b*_2_*b*_3_…*b_n_*).

The evolution of QIEA is conducted through quantum operations on the qubits of individuals in the population. These quantum operations are usually called quantum gates. A quantum gate is a reversible gate represented by a unitary operator *U* acting on the qubit basis states. A unitary operator satisfies *U*^+^*U* = *UU*^+^, where *U*^+^ is the Hermitian adjoint of *U*. Although many quantum gates have been developed for quantum computing, only a quantum rotation gate is used in QIEA to update individuals during the evolution process. The quantum rotation gate *U_R_*(*θ*) is applied to update the probability amplitudes of quantum qubits in each individual to improve the quality of individuals in a population. *U*(*θ_i_*) is defined by a matrix:

(16) $$U_{R}(\theta )=\left[\begin{array}{cc} \cos(\theta ) & -\sin(\theta )\\ \sin(\theta ) & \cos(\theta ) \end{array}\right]$$

where *θ* is the rotation angle.

For example, a qubit $\left[\begin{array}{c} \alpha_{i}\\ \beta_{i} \end{array}\right]$ in an individual can be updated by a quantum rotation gate using

(17) $$\left[\begin{array}{c} \alpha_{i}^{\prime }\\ \beta_{i}^{\prime } \end{array}\right]=U_{R}(\theta_{i})\left[\begin{array}{c} \alpha_{i}\\ \beta_{i} \end{array}\right]$$

where $\left[\begin{array}{c} \alpha_{i}^{\prime }\\ \beta_{i}^{\prime } \end{array}\right]$ is the qubit after quantum rotation gate operation, and *θ_i_* is the rotation angle determined by

(18) $$\theta_{i}=s(\alpha_{i}\beta_{i})\Delta \theta_{i}$$

where *s*(*α_i_β_i_*) is the sign of *θ_i_*, and Δ*θ_i_* is the magnitude of the rotation angle. In the traditional QIEA, Δ*θ_i_* is selected based on experience and experiments. Different Δ*θ_i_* values for different cases are stored in a lookup table [23]. During the evolution process in QIEA, the lookup table is searched to identify the proper value for a specific qubit. Δ*θ_i_* is a key parameter in QIEA that influences computation quality and efficiency. When Δ*θ_i_* is too small, the algorithm may need too many iterations to converge into the optimal solution. When Δ*θ_i_* is too large, the solutions may be trapped into divergent locations or lead to a premature convergence to a local optimum.

### 3.3. The Adaptive Quantum-Inspired Evolutionary Algorithm

In this research, the classic quantum-inspired evolutionary algorithm (QIEA) has been modified to an adaptive quantum-inspired evolutionary algorithm (AQIEA) to improve optimization quality and efficiency.

#### 3.3.1. Adaptive Population Updating Operation

In the classic QIEA, the population is primarily updated by the quantum rotation gate. The values of the rotation angles, however, are very difficult to select. Even though some rotation angles can be selected from the lookup tables, the values of these rotation angles are not changed during the population evolution process. Inspired by Xiong et al. [27], in this research, an adaptive QIEA (AQIEA) with a change of rotation angle in the evolution process is introduced to improve the quality and efficiency of the QIEA.

**Figure 4:**
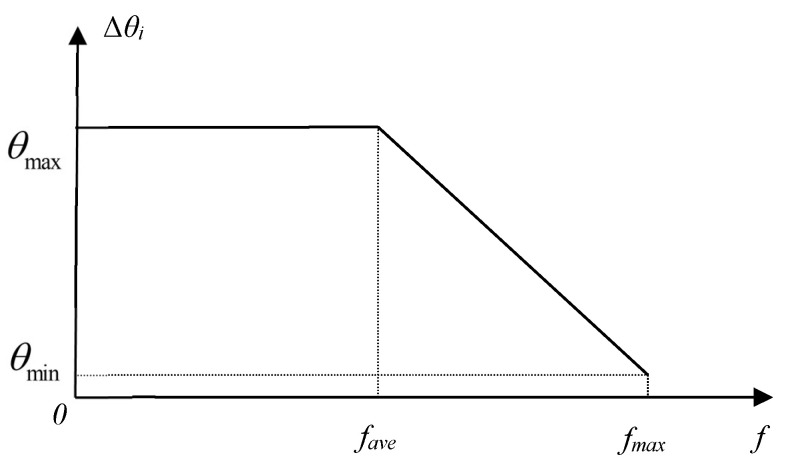
Adaptive change of rotation angle magnitude of quantum rotation gate.

It has been found that when an individual is away from the optimal solution, a large value should be selected for the rotation angle to change the individual quickly towards the optimal solution. When an individual is close to the optimal solution, a small value should be selected for the rotation angle to avoid the individual moving away from the optimal solution. Based on this idea, an adaptive method is introduced in this research to adjust the value of the rotation angle in the optimization process. The magnitude of the rotation angle is calculated by

(19) $$\Delta \theta_{i}=\left\{\begin{array}{ll} \theta_{\max}-(\theta_{\max}-\theta_{\min})\frac{f-f_{ave}}{f_{\max}-f_{ave}} & f>f_{ave}\\ \theta_{\max} & f\leq f_{ave} \end{array}\right.$$

where *θ*_max_ and *θ*_min_ are two positive real numbers (0.001*π* ≤ *θ*_min_ < *θ*_max_ ≤ 0.5*π*). *f*_max_ is the maximum fitness in the current population, *f_ave_* is the average fitness of the individuals in the current population, and *f* is the fitness of the selected individual. Figure 4 illustrates the adaptive change of the rotation angle magnitude of the quantum rotation gate. If the fitness of the selected individual is smaller than or equal to the average fitness of the current population, the magnitude of the rotation angle is selected as a constant *θ*_max_. If the fitness of the selected individual is larger than the average fitness of the current population, the magnitude of the rotation angle is then selected based on the value of *f*. When *f* = *f*_max_, Δ*θ_i_* is selected as *θ*_min_.

The sign of the rotation angle is determined by

(20) $$s(\alpha_{i}\beta_{i})=\left\{\begin{array}{cc} \mathrm{sgn}((o_{i}^{*}-c)\alpha_{i}\beta_{i}) & \alpha_{i}\beta_{i}\neq 0\\ 0 & \alpha_{i}=0\;and\;o_{i}^{*}=1\;or\;\beta_{i}=0\;and\;o_{i}^{*}=0\\ \pm 1 & other \end{array}\right.$$

where sgn(•) is the sign function, o_i_* is the observed state (0 or 1) of the *i*-th qubit of the best individual, and *c* is a positive real number between 0 and 1. Equation (19) can be proved as follows.

**Figure 5:**
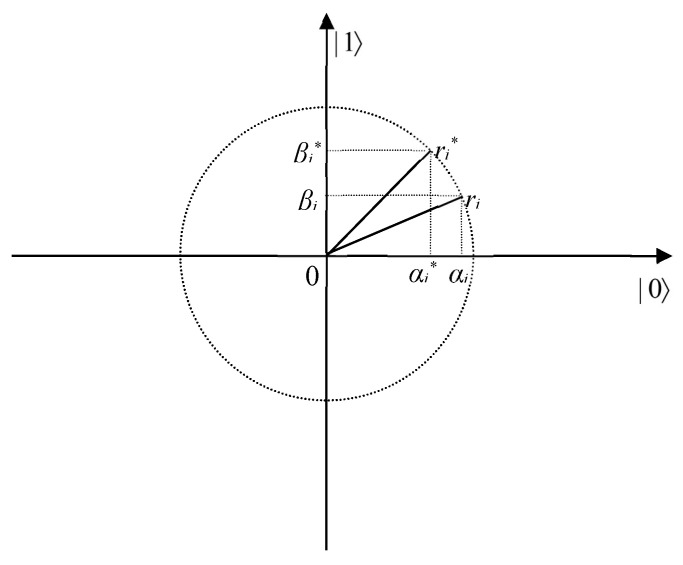
Polar plot of quantum rotation gate for qubit.

**Proof.**  Assume r_i_* is the *i*-th qubit of the best individual, *r_i_* is the *i*-th qubit of the selected individual as shown in Figure 5 in the polar plot, and these qubits are represented as

(21) $$r_{i}^{*}=\left[\begin{array}{c} \alpha_{i}^{*}\\ \beta_{i}^{*} \end{array}\right],\;r_{i}=\left[\begin{array}{c} \alpha_{i}\\ \beta_{i} \end{array}\right]$$

The observed states for the two states r_i_* and *r_i_* are defined by o_i_* and *o_i_*, respectively. □

The possible direction of the rotation angle is decided as follows:

(1) When o_i_* = 0 and *o_i_* = 1: if *α_i_β_i_* > 0, since *r_i_* lies in the I or III quadrant, s(*α_i_β_i_*) should be negative (i.e., −1) to increase the probability amplitudes of $|0\rangle$; if *α_i_β_i_* < 0, since *r_i_* lies in the II or IV quadrant, s(*α_i_β_i_*) should be positive (i.e., +1) to increase the probability amplitudes of $|0\rangle$; if *α_i_* = 0, since *r_i_* lies on the $|1\rangle$ axis, both +1 and −1 of s(*α_i_β_i_*) can increase the probability amplitudes of |0〉, so s(*α_i_β_i_*) is selected as ±1; if *β_i_* = 0, since *r_i_* lies on the $|0\rangle$ axis, the probability amplitudes of $|0\rangle$ are 1, so s(*α_i_β_i_*) is selected as 0.

(2) When o_i_* = 1 and *o_i_* = 0: if *α_i_β_i_* > 0, since *r_i_* lies in the I or III quadrant, s(*α_i_β_i_*) should be +1 to increase the probability amplitudes of $|1\rangle$; if *α_i_β_i_* < 0, since *r_i_* lies in the II or IV quadrant, s(*α_i_β_i_*) should be −1 to increase the probability amplitudes of $|1\rangle$; if *α_i_* = 0, since *r_i_* lies on the $|1\rangle$ axis, the probability amplitudes of |1〉 are 1, so s(*α_i_β_i_*) is selected as 0; if *β_i_* = 0, since *r_i_* lies on the $|0\rangle$ axis, both +1 and −1 of s(*α_i_β_i_*) can increase the probability amplitudes of $|0\rangle$, so s(*α_i_β_i_*) is selected as ±1.

(3) When o_i_* = 0 and *o_i_* = 0: if *α_i_β_i_* > 0, since *r_i_* lies in the I or III quadrant, s(*α_i_β_i_*) should be negative (i.e., −1) to increase the probability amplitudes of $|0\rangle$; if *α_i_β_i_* < 0, since *r_i_* lies in the II or IV quadrant, s(*α_i_β_i_*) should be positive (i.e., +1) to increase the probability amplitudes of $|0\rangle$; if *α_i_* = 0, since *r_i_* lies on the $|1\rangle$ axis, both +1 and −1 of s(*α_i_β_i_*) can increase the probability amplitudes of $|0\rangle$, so s(*α_i_β_i_*) is selected as ±1; if *β_i_* = 0, since *r_i_* lies on the $|0\rangle$ axis, the probability amplitudes of $|0\rangle$ are 1, so s(*α_i_β_i_*) is selected as 0.

(4) When o_i_* = 1 and *o_i_* = 1: if *α_i_β_i_* > 0, since *r_i_* lies in the I or III quadrant, s(*α_i_β_i_*) should be +1 to increase the probability amplitudes of $|1\rangle$; if *α_i_β_i_* < 0, since *r_i_* lies in the II or IV quadrant, s(*α_i_β_i_*) should be −1 to increase the probability amplitudes of $|1\rangle$; if *α_i_* = 0, since *r_i_* lies on the $|1\rangle$ axis, the probability amplitudes of $|1\rangle$ are 1, so s(*α_i_β_i_*) is selected as 0; if *β_i_* = 0, since *r_i_* lies on the $|0\rangle$ axis, both +1 and −1 of s(*α_i_β_i_*) can increase the probability amplitudes of $|0\rangle$, so s(*α_i_β_i_*) is selected as ±1.

Table 1 shows the summary based on the above discussions. It is found from Table 1 that the sign of the rotation angle is determined based on o_i_*, *α_i_*, and *β_i_*, but not *o_i_*. This is because when o_i_* = 1, the probability amplitudes of $|1\rangle$ should be increased, no matter if the value of *o_i_* is 0 or 1. Similarly, when o_i_* = 0, the probability amplitudes of $|0\rangle$ should be increased, no matter if the value of *o_i_* is 0 or 1.

The summary given in Table 1 can be simplified to the summary shown in Table 2. The sign in Equation (18) is obtained based on Table 2. For example, if *α_i_β_i_* > 0 and o_i_* = 0, the *s*(*α_i_β_i_*) is obtained as −1 according to Table 2.

#### 3.3.2. Adaptive Mutation Operation

To avoid being trapped into local optimal locations and to improve global searching ability, an adaptive quantum mutation operation is introduced in this work in the AQIEA. For a selected individual in the population, the mutation operation is conducted based on a mutation probability:

(22) $$p_{m}=\left\{\begin{array}{ll} p_{\max}-(p_{\max}-p_{\min})\frac{f-f_{ave}}{f_{\max}-f_{ave}} & if\;f>f_{ave}\\ p_{\max} & f\leq f_{ave} \end{array}\right.$$

where *p*_max_ and *p*_min_ are two small positive real values between 0 and 1. When an individual is selected for mutation, two random positions in the individual are selected and the two qubits are mutated by a quantum-not gate. A quantum-not gate *U_X_* is defined by a matrix:

(23) $$U_{X}=\left[\begin{array}{cc} 0 & 1\\ 1 & 0 \end{array}\right]$$

For example, a qubit $\left[\begin{array}{c} \alpha_{i}\\ \beta_{i} \end{array}\right]$ in an individual can be mutated by a quantum-not gate through

(24) $$\left[\begin{array}{c} \alpha_{i}^{\prime }\\ \beta_{i}^{\prime } \end{array}\right]=U_{X}\left[\begin{array}{c} \alpha_{i}\\ \beta_{i} \end{array}\right]=\left[\begin{array}{c} \beta_{i}\\ \alpha_{i} \end{array}\right]$$

where $\left[\begin{array}{c} \alpha_{i}^{\prime }\\ \beta_{i}^{\prime } \end{array}\right]$ is the qubit after quantum-not gate operation. Obviously, the quantum-not gate operation is used to swap the probability amplitudes of the two quantum states $|0\rangle$ and $|1\rangle$.

#### 3.3.3. The Procedures in AQIEA

With the proposed adaptive quantum rotation and mutation operations, the proposed quantum-inspired evolutionary algorithm can effectively enhance the population diversity and further accelerate the convergence speed and avoid being trapped in the local optimal. The detail procedures in AQIEA are described as follows (Procedure 1).
**Procedure 1.** Procedure of AQIEA.
**Begin Proc**(1)Set *t* = 0, initialize the population *Q*(*t*) by assigning each pair of *αi* and *β_i_* in each individual in *Q*(*t*) as $1/\sqrt{2}$ , representing that each qubit has the same probabilities to be observed as |0〉 and |1〉 at the initialization step;(2)Create a set of binary strings *P*(*t*) for representing the observing states of *Q*(*t*) based on the **Procedure 2 (Procedure of decoding)**;
(3)Evaluate all binary strings in *P*(*t*);(4)Store the best solution among all binary strings in *P*(*t*) as P*;(5)**While** (termination condition is not satisfied) **do**
(i)Increase *t*’s value by *t* = *t* + 1;(ii)Create a set of binary strings *P*(*t*) for representing the observing states of *Q*(*t*) based on the **Procedure 2;**(iii)Evaluate all binary strings in *P*(*t*);(iv)Select the best solution among all binary strings in *P*(*t*), and compare it with the P*. If this best solution is better than P*, replace P* with this best solution; otherwise, keep P* unchanged;(v)Update individuals in *Q*(*t*) with the quantum rotation gate based on the **Procedure 3 (Procedure of population updating)**;(vi)Apply mutation operation to individuals in *Q*(*t*) based on the **Procedure 4 (Procedure of mutation operation)**.  **End while** **End Proc**

The decoding of a quantum individual into a classic bit individual is conducted based on the following Procedure 2.
**Procedure 2.** Procedure of decoding.
**Begin Proc** **For** (**each** *q_j_*(*t*) **in** *Q*(*t*))(1)Initialize a binary string *P_j_*(*t*) as a null list for representing the observing state of *q_j_*(*t*); initialize a decimal list *C_j_*(*t*) as a null list for representing the selected values of parameters in optimization solution in the individual;(2)**For** (**each** pair of *α_i_* and *β_i_* in *q_j_*(*t*))
(i)Generate a random number *r* between 0 and 1;(ii)If *r* ≥ |*α_i_*|^2^, the corresponding observing state of the qubit is 1; otherwise, the observing state is 0. Add the observing state to *P_j_*(*t*).  **End for**
(3)Convert the binary string *P_j_*(*t*) to a decimal list *C_j_*(*t*) with values of parameters in an optimization solution based on Equation (15).**End for** **End Proc**

The adaptive population updating operation is conducted using the following Procedure 3.
**Procedure 3.** Procedure of population updating.
**Begin Proc** **For** (**each** *q_j_*(*t*) **in** *Q*(*t*)) **For** (**each** pair of *α_i_* and *β_i_* **in** *q_j_*(*t*))(i)Calculate Δ*θ_i_* and *s* (*α_i_β_i_*) based on Equations (19) and (20), respectively;(ii)Update *α_i_* and *β_i_* based on Equations (16)–(18).  **End for** **End for** **End Proc**

The adaptive mutation operation is conducted using the following Procedure 4.
**Procedure 4.** Procedure of mutation operation.**Begin Proc** **For** (**each** *q_j_*(*t*) **in** *Q*(*t*))(1)Generate a random number *r* between 0 and 1;(2)If *r* < *p_m_*, where *p_m_* is calculated from Equation (22), generate two random mutation positions *p*_1_ and *p*_2_ in *q_j_*(*t*). If *p*_1_ ≠ *p*_2_, conduct mutation operations to the qubits in positions of *p*_1_ and *p*_2_ based on Equation (24); otherwise, do nothing;(3)Otherwise, do nothing.**End for** **End Proc**

## 4. Identification of the Optimal Calibration Parameters for Dead Reckoning Based on the AQIEA

### 4.1. Experimental Setup for Dead Reckoning

**Figure 6:**
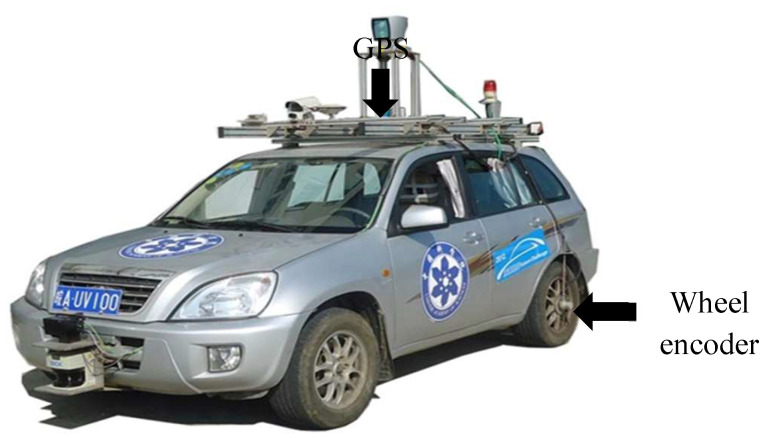
“Intelligent Pioneer II” intelligent vehicle experimental platform.

The intelligent vehicle experimental platform, “Intelligent Pioneer II” (Figure 6) developed by the Intelligent Vehicle Research Center, Hefei Institutes of Physical Science (HIPS), was used to carry out the dead reckoning experiments. Two wheel encoders that were mounted on the two rear wheels of the vehicle were used to provide the odometry readings, and the DGPS sensor (NovAtel SPAN-CPT), a tightly coupled GPS/INS integrated navigation system, was used to provide real-time positioning information for calculation of the localization errors and to initialize the dead reckoning system.

The nominal parameters of the vehicle and the sensors are given in Table 3.

### 4.2. Identification of the Optimal Dead Reckoning Calibration Parameters Based on the AQIEA

**Figure 7:**
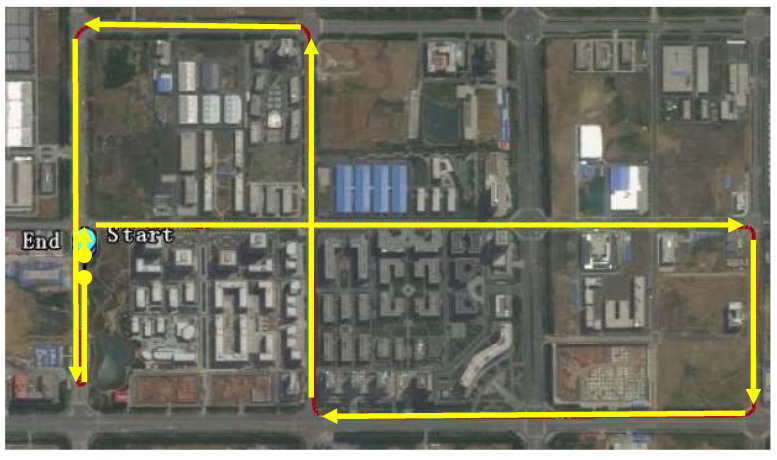
The selected calibration path.

It should be noted that unlike traditional DR calibration methods such as UMBmark [4], the calibration path in this work was selected from normal suburb roads. Because the considered optimization parameters for the calibration are the compensation values to the left rear wheel, right rear wheel, and vehicle heading, the designed calibration path should contain enough scenarios (including both left and right turns) and enough mileage. To calibrate the DR, a calibration path needs to be selected as shown in Figure 7. The yellow arrows in the figure mean the driving directions and route. The total length of the calibration path was about 6 km.

During the experiment, the vehicle was driven along the selected path, and the positioning data (i.e., longitude, latitude, and heading) of the vehicle from the DGPS and the pulse readings from the two wheel encoders and the gyroscope were recorded simultaneously. Because the two different kinds of data from the DGPS and the DR were in different coordinate systems (i.e., DGPS in the WGS84 coordinate system and DR in the Cartesian coordinate system), these data had to be converted into the same coordinate system. In this work, the longitude and latitude from the DGPS were converted into the Cartesian coordinate system by

(25) $$\left\{\begin{array}{l} x_{t}^{gps}=R\cos(\varphi_{t}^{m})\Delta \lambda_{t}\\ y_{t}^{gps}=R\Delta \varphi_{t} \end{array}\right.$$

where *R* = 6,371,004 m is the radius of the earth, $\left(x_{t}^{gps},y_{t}^{gps}\right)$ is the vehicle’s position at time *t* in the Cartesian coordinate system, and Δ*λ_t_* and Δ*φ_t_* are the differences of the longitude and latitude between the initial position and the position at time *t*, respectively. Δ*λ_t_*, Δ*φ_t_*, and *φ_t_^m^* are defined by

(26) $$\left\{\begin{array}{l} \Delta \lambda_{t}=\lambda_{t}-\lambda_{0}\\ \Delta \varphi_{t}=\varphi_{t}-\varphi_{0}\\ \varphi_{t}^{m}=\frac{\varphi_{t}+\varphi_{0}}{2} \end{array}\right.$$

where (*λ*_0_, *φ*_0_) and (*λ_t_*, *φ_t_*) are the initial position and the position at time *t* in the WGS84 coordinate system, respectively. The units of *λ*_0_, *φ*_0_, *λ_t_*, and *φ_t_* are radians.

After the data were collected, the maximum error and mean error considering all the positions were selected as the objective functions to build the two optimization models. For each optimization model, each of the three calibration parameters (i.e., *c*_1_, *c*_2_, and *c*_3_) was encoded by 20 qubits and so each individual was encoded by 60 qubits, the AQIEA was run 10 times, and the termination condition for the two optimization models was set as 10,000 generations.

For the first optimization model to minimize the maximum error, the optimal calibration parameters were obtained as: *c*_1_ = 0.642 m, *c*_2_ = −0.455 m, and *c*_3_ = 0.377°. The maximum positioning error was obtained as 4.7398 m. For the second optimization model to minimize the mean error, the optimal calibration parameters were obtained as: *c*_1_ = 0.177 m, *c*_2_ = 0.020 m, and *c*_3_ = 0.425°. The mean positioning error was obtained as 1.6838 m.

**Figure 8:**
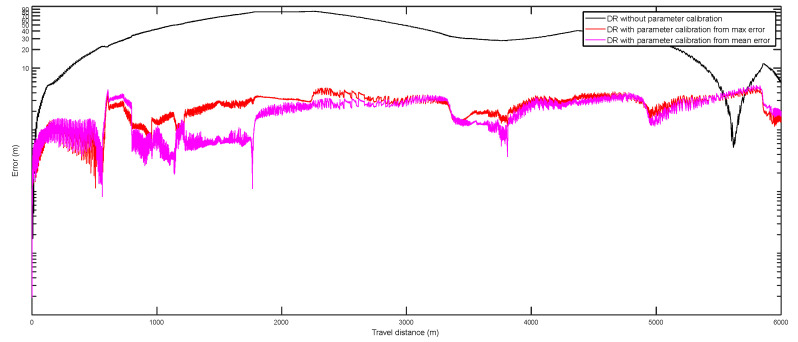
Errors along the test path for the three different DR localization methods.

Figure 8 (the *y*-axes are plotted with logarithmic scale) gives the errors along the selected calibration path for the three different DR positioning datasets, i.e., DR without parameter calibration, DR with parameter calibration through minimization of the maximum error, and DR with parameter calibration through minimization of the mean error. From the figure, it is can be found that, compared to the result of DR without parameter calibration, the DR with the parameter calibration through two different optimization models can improve the positioning accuracy significantly. From the figure, it can also be seen that the DR data with the parameter calibration through minimization of the mean error can provide the best results. Thus, the calibration parameters obtained through minimization of the mean error were selected as the final calibration parameters.

### 4.3. Experiments for Dead Reckoning with Error Compensation

Two different dead reckoning experiments with different scales of the road paths were conducted to evaluate the effectiveness of the developed calibration scheme. The first road path was about 16 km, and the second one was about 43 km. In these two experiments, the same calibration parameters that were obtained as explained in Section 4.2 were used in the dead reckoning model, and the DGPS was just used for obtaining the headings of the vehicle and the error calculations. The DR self-localization was conducted in real time during the driving processes.

**Figure 9:**
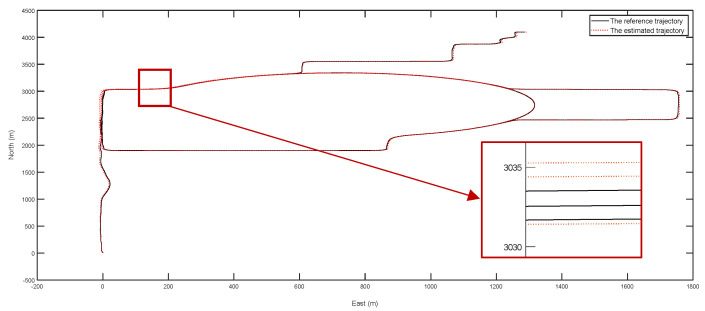
The reference trajectory and estimated trajectory of the first test (about 16 km).

**Figure 10:**
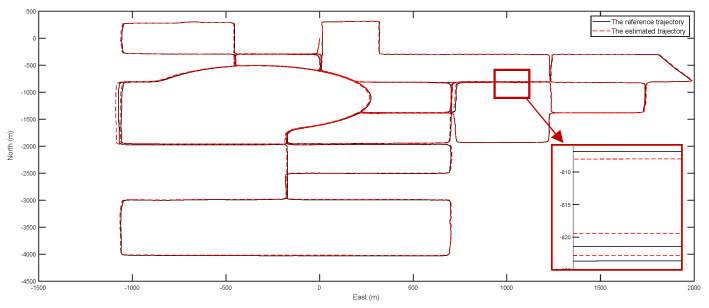
The reference trajectory and estimated trajectory of the second test (about 43 km).

The reference trajectory (DGPS) and estimated trajectory for the two tests are given in Figure 9 and Figure 10, respectively. During the two experiments, some road segments were repeated twice to test the repeatability of the positioning accuracy (which is similar to the closure error) of the calibrated dead reckoning-based positioning method. From the experimental results, it was found that the maximum repeated error of positioning accuracy was about 1 m for the 16 km test and 3 m for the 43 km test.

**Figure 11:**
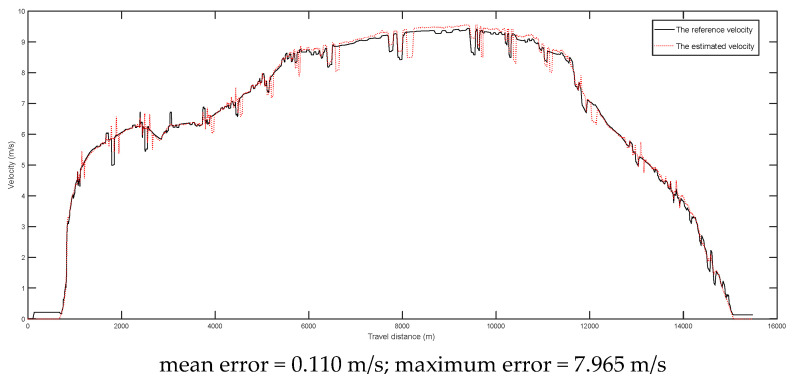
The comparison between reference velocities and estimated velocities during the first test (about 16 km).

**Figure 12:**
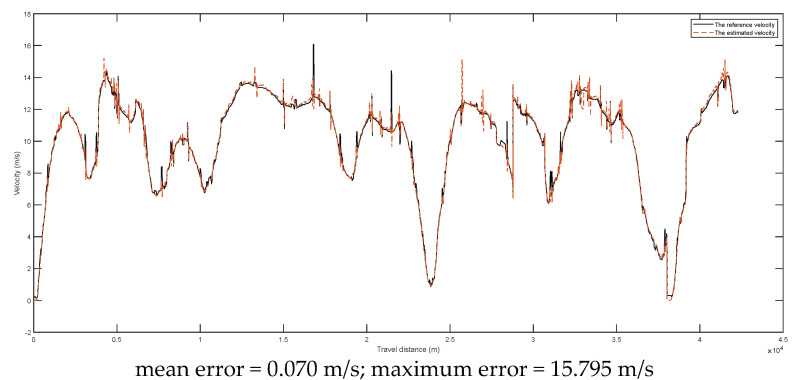
The comparison between reference velocities and estimated velocities during the second test (about 42 km).

The comparison between the reference velocities (DGPS) and the estimated velocities during the two tests are given in Figure 11 and Figure 12, respectively. From the results, it was found that the mean velocity errors were reduced to 0.110 m/s and 0.070 m/s for the tests of 16 km and 43 km, respectively. It should be noted that the large maximum errors were due to the random noises.

**Figure 13:**
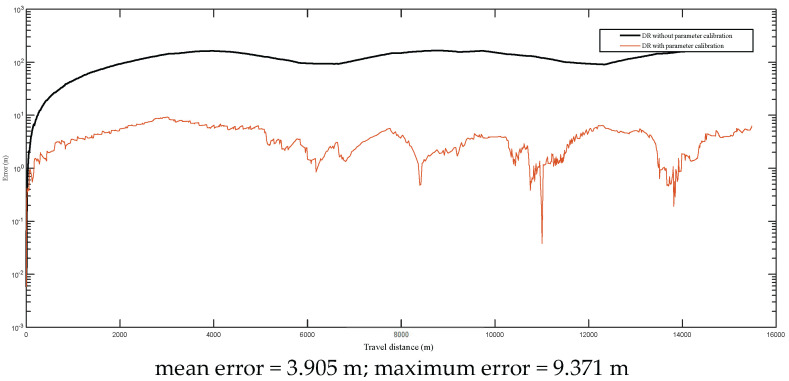
The positional errors of the first test (about 16 km).

**Figure 14:**
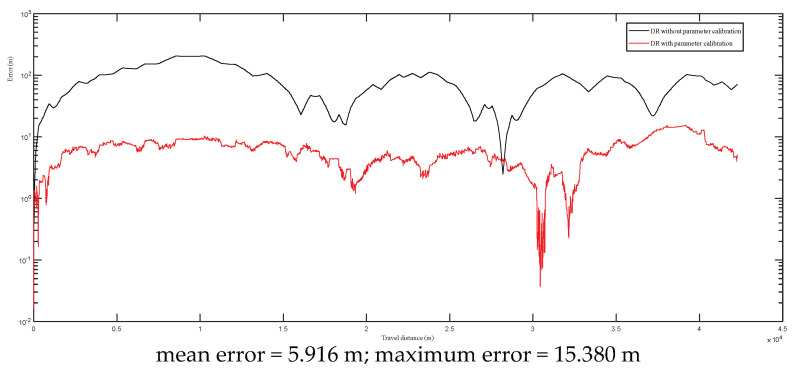
The positional errors of the second test (about 43 km).

The positional errors are given in Figure 13 and Figure 14 (the *y*-axes are plotted with logarithmic scale), respectively. From the experimental results, it can be found that the maximum errors reached up to 200 m during the 16 km and 42 km field tests. After the calibrated parameters were applied, the maximum and mean errors were reduced significantly. For the 16 km test, its mean error and maximum error were reduced to 3.905 m and 9.371 m, respectively. For the 42 km test, its mean error and maximum error were reduced to 5.916 m and 15.380 m, respectively. It should be noted that the curve shapes of DR errors follow different patterns due to the change of the vehicle’s driving direction. When the driving direction is not changed, the DR errors may be increased continuously. When the driving direction is changed or driving on driving on a trajectory where there are loop routes, such as the selected test trajectories in this study (e.g., Figure 7, Figure 9 and Figure 10), the DR errors can be reduced. The detailed analysis can be consulted in reference [4].

From the above experimental results, it can be concluded that the developed method can be used to calibrate the dead reckoning parameters to reduce the systematic errors effectively. With this method, the dead reckoning can be used as an alternative high accuracy localization tool when the GPS is out of service.

## 5. Comparative Study for the AQIEA

### 5.1. Benchmark Functions

To further demonstrate the quality and efficiency of the newly developed AQIEA optimization method, the AQIEA was compared with the classic genetic algorithm (CGA) [28] and the classic quantum-inspired evolutionary algorithm (CQIEA) [23]. Eight benchmark functions were selected to evaluate the performance of these three algorithms. The search spaces of the first four functions (*f*_1_~*f*_4_) are 2-dimensional and the search spaces of the other four functions (*f*_5_~*f*_8_) are 30-dimensional. Table 4 gives the detailed descriptions of the eight benchmark functions. In this table, D is the dimension of the search space, R is the value range of each variable, and O is the optimal value under the given constraints.

### 5.2. Results for Comparative Study

To test these benchmark functions, the specific parameters for the three algorithms must be given. Table 5 provides parameter settings for these three algorithms. It should be noted that to keep the manuscript more concise, the quantum rotation angles in CQIEA were omitted and can be consulted in reference [23]. Because the complexities of the eight functions are different, different maximum iterations were selected to obtain the best results. In this comparative study, the maximum generations were selected as 5000 for functions *f*_1_~*f*_4_, and the maximum generations were selected as 10,000 for functions *f*_5_~*f*_8_.

All the three algorithms were implemented in MATLAB running on a computer with the Intel Core i3-3129M (dual cores 2.4G) CPU and the RAM of 2 GB. Due to the stochastic nature of the evolutionary algorithm, each algorithm was run 30 times for each benchmark function. Table 6 gives the experimental results of the three algorithms. In this table, the best value, the worst value, the mean value, the standard deviation, and the mean computation time considering the 30 runs for each of the test function using each of the evolutionary algorithms are provided.

From Table 6, we can see that the adaptive quantum-inspired evolutionary algorithm (AQIEA) developed in this research nearly provides the best computation quality and efficiency among the selected three algorithms. The AQIEA takes the shortest time because it needs a smaller population size than CGA to achieve the optimization results, and unlike CQIEA, it does not need to search the lookup table for conducting the quantum rotation operations.

From the experimental results, it can be found that by applying the adaptive quantum rotation operation and the adaptive quantum mutation operation, the developed AQIEA can effectively prevent the optimization results from being trapped into divergent locations or premature convergence to local optimums. Due to the search ability of the proposed algorithm, it is very effective at optimizing the error function for dead reckoning to obtain optimal calibration parameters.

## 6. Conclusions

The developed method aims at reducing systematic errors of dead reckoning-based positioning for intelligent vehicles. In this study, an adaptive quantum-inspired evolutionary algorithm-based dead reckoning calibration scheme was proposed. Real road experiments were conducted to evaluate the effectiveness of the developed method and the experiment results show that the maximum error and average error of dead reckoning can be reduced significantly. With this method, the dead reckoning can be used as an alternative high accuracy localization tool when the GPS is out of service. Advantages of the developed method are summarized as follows:

(1) The newly developed dead reckoning positioning parameter calibration scheme is effective at compensating the systematic errors in dead reckoning. In addition, the design of a special calibration path is not required in this method for the calibration experiment.
(2) The developed adaptive quantum-inspired evolutionary algorithm is effective at identifying the optimal calibration parameters for the dead reckoning.
(3) The adaptive quantum rotation operation and the adaptive quantum mutation operation introduced in this research are effective at improving the computation quality and efficiency of the quantum-inspired evolutionary algorithm.

The developed calibration method was only tested on paved road with a smooth surface and only systematic errors of dead reckoning were considered. Additional tests will be conducted on different road conditions and an improved method which considers both systematic errors and random errors will be developed in the future. Moreover, for the proposed adaptive quantum-inspired evolutionary algorithm, a multi-objective version will be developed in the future.

## Figures and Tables

**Table 1:** Summary of signs for the rotation angle.

*o_i_*	o_i_*	*s*(*α_i_β_i_*)			
		*α_i_β_i_* > 0	*α_i_β_i_* < 0	*α_i_* = 0	*β_i_* = 0
1	0	−1	+1	±1	0
0	1	+1	−1	0	±1
0	0	−1	+1	±1	0
1	1	+1	−1	0	±1

**Table 2:** Simplified summary of signs for the rotation angle.

o_i_*	*s*(*α_i_β_i_*)			
	*α_i_β_i_* > 0	*α_i_β_i_* < 0	*α_i_* = 0	*β_i_* = 0
0	−1	+1	±1	0
1	+1	−1	0	±1

**Table 3:** The nominal parameters of the vehicle and the sensors.

Parameter	Name	Value
*R_r_*	Resolution of encoder for rear right wheel	4000 pulses/r
*R_l_*	Resolution of encoder for rear left wheel	4000 pulses/r
*D_r_*	Nominal diameter of right rear wheel	0.637 m
*D_l_*	Nominal diameter of left rear wheel	0.637 m

**Table 4:** Benchmark test functions.

No.	Function	Formulation	D	R	O
*f*_1_	De-Jong’s function	$f_{1}=100{\left(x_{1}^{2}-x_{2}\right)}^{2}+{\left(1-x_{1}\right)}^{2}$	2	[−2.048, 2.048]	0
*f*_2_	Goldstein–Price function	$\begin{array}{l} f_{2}=[1+{\left(x_{1}+x_{2}+1\right)}^{2}(19-14x_{1}+\\ \;\;3x_{2}^{2}-14x_{2}+6x_{1}x_{2})+3x_{2}^{2}]\\ \;\;[30+{\left(2x_{1}-3x_{2}\right)}^{2}(18-32x_{1}+\\ \;\;12x_{1}^{2}+48x_{2}-36x_{1}x_{2}+27x_{2}^{2})] \end{array}$	2	[−100, 100]	3
*f*_3_	Schaffer’s F6 function	$f_{3}=0.5+\frac{\sin^{2}\sqrt{x_{1}^{2}+x_{2}^{2}}-0.5}{{\left[1+0.001(x_{1}^{2}+x_{2}^{2})\right]}^{2}}$	2	[−100, 100]	0
*f*_4_	Easom’s function	$\begin{array}{l} f_{4}=-\cos(x_{1})\cos(x_{2})\\ \;\;\exp[-{\left(x_{1}-\pi \right)}^{2}-{\left(x_{2}-\pi \right)}^{2}] \end{array}$	2	[−100, 100]	−1
*f*_5_	Sphere function	$f_{5}=\sum_{i=1}^{n}x_{i}^{2}$	30	[−5.12, 5.12]	0
*f*_6_	Griewank function	$f_{6}=\frac{1}{4000}\sum_{i=1}^{n}x_{i}^{2}-\prod_{i=1}^{n}\cos(\frac{x_{i}}{\sqrt{i}})+1$	30	[−600, 600]	0
*f*_7_	Ackley function	$\begin{array}{l} f_{7}=-20\exp(-0.2\sqrt{\frac{1}{n}\sum_{i=1}^{n}x_{i}^{2}})-\\ \;\;\exp[\frac{1}{n}\sum_{i=1}^{n}\cos(2\pi x_{i})]+20+e \end{array}$	30	[−32, 32]	0
*f*_8_	Rastrigin function	$f_{8}=\sum_{i=1}^{n}\left[x_{i}^{2}-10\cos(2\pi x_{i})+10\right]$	30	[−5.12, 5.12]	0

**Table 5:** Main parameters for comparing the three algorithms.

Parameter	CGA	CQIEA	AQIEA
Population size	60	10	10
Maximum generation	5000 for *f*_1_~*f*_4_ 10,000 for *f*_5_~*f*_8_	5000 for *f*_1_~*f*_4_ 10,000 for *f*_5_~*f*_8_	5000 for *f*_1_~*f*_4_ 10,000 for *f*_5_~*f*_8_
Crossover rate	0.7	/	/
Mutation rate	0.01	/	*p_max_* = 0.1, *p_min_* = 0.01
Quantum rotation angle	/	Given in [23]	*θ_max_* = 0.05*π*, *θ_min_* = 0.001*π*

**Table 6:** Experimental results of the three algorithms.

Test Function	Performance	Algorithms		
		AQIEA	CGA	CQIEA
*f*_1_	Best value	**1.0004 × 10^−10^**	1.0004 × 10^−10^	8.2224 × 10^−8^
	Worst value	**8.0830 × 10^−2^**	1.9749 × 10^−1^	1.0000
	Mean value	**7.3313 × 10^−3^**	5.0742 × 10^−2^	7.9007 × 10^−2^
	Standard deviation	**1.7405 × 10^−2^**	6.4377 × 10^−2^	2.5115 × 10^−1^
	Mean time (s)	**15.8**	30.2	25.3
*f*_2_	Best value	**3.0000**	3.0000	3.0000
	Worst value	**8.4600 × 10^1^**	5.7482 × 10^5^	6.1677 × 10^7^
	Mean value	**9.0448**	1.9191 × 10^4^	2.0560 × 10^6^
	Standard deviation	**1.6197 × 10^1^**	1.0494 × 10^5^	1.1261 × 10^7^
	Mean time (s)	**13.9**	30.4	28.8
*f*_3_	Best value	**1.8190 × 10^−8^**	1.8190 × 10^−8^	1.8190 × 10^−8^
	Worst value	**9.6583 × 10^−3^**	1.2439 × 10^−1^	1.2439 × 10^−1^
	Mean value	**5.9255 × 10^−3^**	1.4411 × 10^−2^	1.0522 × 10^−2^
	Standard deviation	**4.5687 × 10^−3^**	3.0204 × 10^−2^	2.1957 × 10^−2^
	Mean time (s)	**19.9**	57.2	25.8
*f*_4_	Best value	**−1.0000**	−1.0000	−1.0000
	Worst value	**−1.0000**	−4.6552 × 10^−7^	0.0000
	Mean value	**−1.0000**	−9.6631 × 10^−1^	−3.3333 × 10^−2^
	Standard deviation	**0.0000**	1.8251 × 10^−1^	1.8257 × 10^−1^
	Mean time (s)	**13.6**	30.0	28.9
*f*_5_	Best value	**1.6051 × 10^−5^**	4.1621	1.2827 × 10^−4^
	Worst value	**2.5806 × 10^−2^**	7.3858	2.1318 × 10^−1^
	Mean value	**1.3603 × 10^−3^**	5.8291	7.9335 × 10^−3^
	Standard deviation	**4.6669 × 10^−3^**	8.3242 × 10^−1^	3.8769 × 10^−2^
	Mean time (s)	**277.2**	374.4	637.2
*f*_6_	Best value	**6.2675 × 10^−5^**	1.6512 × 10^1^	1.9451 × 10^2^
	Worst value	**1.8752 × 10^−5^**	2.6707 × 10^1^	9.4725 × 10^1^
	Mean value	**4.6470 × 10^−5^**	2.2313 × 10^1^	3.4142 × 10^1^
	Standard deviation	**4.5904 × 10^−5^**	2.3307	2.1200 × 10^1^
	Mean time (s)	**270.5**	382.6	614.7
*f*_7_	Best value	**3.3597 × 10^−1^**	9.0554	9.99160
	Worst value	**4.3268**	1.0766 × 10^1^	1.9265 × 10^1^
	Mean value	**2.1113**	1.0020 × 10^1^	1.3341 × 10^1^
	Standard deviation	7.8542 × 10^−1^	**4.2962 × 10^−1^**	2.5355
	Mean time (s)	**347.7**	373.9	657.9
*f*_8_	Best value	**8.1957**	4.0974 × 10^1^	6.6567 × 10^1^
	Worst value	**2.4041 × 10^1^**	8.5433 × 10^1^	8.7187 × 10^1^
	Mean value	**1.3444 × 10^1^**	6.2210 × 10^1^	7.9928 × 10^1^
	Standard deviation	**3.9940**	1.1466 × 10^1^	5.1744
	Mean time (s)	**328.6**	356.7	643.1

## Data Availability

Not applicable.
